# Gut Microbiota and Sleep Disorders with a Special Focus on the Pediatric Population

**DOI:** 10.3390/pediatric18040094

**Published:** 2026-07-11

**Authors:** Alberto Verrotti, Virginia Filippini, Barbara Federici, Valentina Biagioli, Lino Nobili, Pietro Ferrara, Pasquale Striano

**Affiliations:** 1 Department of Pediatrics, University of Perugia, 06129 Perugia, Italy; virginia.filippini@libero.it; 2 Department of Gastroenterology, Hepatology, and Digestive Endoscopy, University of Perugia, 06129 Perugia, Italy; barbara.federici@unipg.it; 3 Department of Neurosciences, Rehabilitation, Ophthalmology, Genetics, Maternal and Child Health, University of Genoa, 16126 Genoa, Italy; valentina.biagioli@edu.unige.it (V.B.); lino.nobili@unige.it (L.N.); strianop@gmail.com (P.S.); 4 IRCCS Istituto Giannina Gaslini, 16147 Genoa, Italy; 5 Unit of Pediatrics, Campus Biomedico, 00128 Rome, Italy; p.ferrara@policlinicocampus.it

**Keywords:** gut microbiota, sleep disorders, gut–brain axis, dysbiosis, pediatric age

## Abstract

Growing evidence indicates a bidirectional relationship between the gut microbiota and sleep disturbances in children, with the microbiota–gut–brain axis (MGBA) mediating this interaction. Sleep, circadian rhythms, and the gut microbiota form an interdependent and developmentally dynamic network that plays a crucial role in neurodevelopment during infancy and childhood. Although the mechanisms underlying this complex interaction have not yet been fully elucidated, emerging evidence suggests that multiple dimensions of sleep—including duration, quality, timing, and regularity—are closely associated with gut microbial composition and function. These findings support the rationale for nutritional and microbiota-targeted interventions during critical developmental windows. However, most mechanistic and taxonomic evidence derives from adult or mixed-age cohorts, while methodological heterogeneity, geographic bias, and the predominance of cross-sectional studies limit causal inference. This review provides an overview of the recent literature investigating the role of the gut microbiota in sleep and sleep disorders in children and summarizes potential microbiota-based therapeutic strategies.

## 1. Introduction

The gut–brain axis, a communication network between the gastrointestinal tract and the central nervous system, is a well-established system involved in physiological homeostasis and numerous neurological disorders [1]. Moreover, sleep, circadian rhythms, and the gut microbiota form an interdependent, developmentally dynamic network that plays an important role in neurodevelopment during infancy and childhood [2]. Early-life sleep consolidation supports synaptic pruning, memory consolidation, attention, and emotional regulation. At the same time, the maturing gut microbiome produces metabolites, immune signals, and neuroactive compounds that modulate brain development and host circadian biology. The mechanisms of this complex interaction are not yet fully understood. Emerging human evidence links multidimensional sleep health (duration, quality, timing, regularity) to microbial composition and function. Experimental and observational studies also suggest that alterations in microbial taxa and short-chain fatty acid (SCFA) production may represent potential mediators of sleep–brain interactions [3,4,5].

Two complementary syntheses of the human literature highlight convergent signals relevant to pediatrics. A lifespan-focused systematic review [2] found heterogeneous but recurring associations between sleep disturbance/circadian misalignment and changes in *Oscillospiraceae*/*Ruminococcaceae* (notably *Faecalibacterium*) and in microbial metabolic pathways including SCFA, amino-acid, bile-acid, and vitamin-related functions. More recently, a review focusing on the first 1000 days of life highlighted [4] the perinatal and infant windows as uniquely plastic: feeding mode (breastfeeding, HMOs), nutrient exposures (DHA, choline, iron, iodine, B12, vitamin D, folate), antibiotics, socioeconomic factors, geographic variation, and mode of delivery shape microbial maturation and, in several trials, influence infant sleep consolidation and early neurocognitive markers. Therefore, nutrition- and microbiota-targeted interventions may represent promising and modifiable strategies, probably during a critical pediatric window. These factors likely contribute to the heterogeneity observed across studies. Therefore, this heterogeneity should be taken into account when interpreting these findings.

Methodological heterogeneity and the presence of numerous confounding factors represent major limitations in interpreting the current evidence on the association between the gut microbiota and sleep in the pediatric population. Therefore, it is important to take into consideration the main confounding factors. Firstly, diet is a key determinant of gut microbiota composition. Breastfed infants typically exhibit a predominance of *Bifidobacterium*, whereas formula feeding is associated with increased microbial richness. Perinatal and early-life exposure to antibiotics has been shown to substantially alter the functional profile of the gut microbiome for at least the first year of life. Markovic et al. [6] reported that indirect antibiotic exposure during childbirth was associated with a higher day-to-night sleep ratio and fewer nighttime awakenings in infants, suggesting a potential microbiota-mediated link between perinatal antibiotic exposure and sleep regulation. Socioeconomic status (SES) has also been identified as an important determinant of gut microbiota composition from early childhood. Ratcliff et al. [7] reported that SES predicts both alpha- and beta-diversity, although the direction of this association varies by setting: in high-income countries, greater microbial diversity is generally observed among socioeconomically advantaged groups, whereas the opposite pattern has been reported in low- and middle-income countries. Similarly, De Filippo et al. [8] demonstrated that children of the same ethnicity living in rural and urban environments (Burkina Faso and Italy) exhibit markedly different gut microbial profiles, highlighting the influence of environmental exposures and dietary patterns on microbiota composition. Mode of delivery also plays a critical role in early microbiome development. Cesarean section, by reducing the vertical transmission of maternal microorganisms and delaying microbiome maturation, significantly alters neonatal microbial composition. It has been associated with alterations in alpha-diversity during early life [9] and with a 57% lower abundance of *Bifidobacterium* compared with vaginal delivery [10]. To improve the reproducibility and comparability of future studies, greater methodological standardization is needed. Recommended strategies include the adoption of consensus reporting guidelines, harmonization of microbiome sampling and analytical protocols, comprehensive assessment of potential confounding factors, and the implementation of longitudinal study designs involving larger and more diverse populations.

In adults with insomnia, a meta-analysis demonstrates reduced observed species and other richness metrics (large effect for observed species), consistent beta-diversity separation, and reproducible taxonomic patterns: decreased *Faecalibacterium* and *Lachnospira* and increased *Blautia* and *Eubacterium hallii* [11]. These taxa correlate with insomnia severity and with inflammatory and metabolomic markers (e.g., *Faecalibacterium* inverse with IL 6; *Blautia* positive with IL 6; phenol/phenol-sulfate and adenosine associations), supporting mechanistic pathways involving reduced SCFA production, altered tryptophan/serotonin–melatonin metabolism, intestinal barrier dysfunction, and immune/HPA-axis activation.

Collectively, these data position early-life sleep and nutritional/microbiome modulation as interventional targets for pediatric neurodevelopment. However, most mechanistic and taxonomic evidence derives from adult or mixed-age cohorts, and methodological heterogeneity, geographic bias, and predominantly cross-sectional designs limit causal inference. For pediatrics, among the others, there are two crucial points: (1) to determine whether the insomnia-associated microbial signatures and functional disturbances are present, modifiable and predictive in infants and children; (2) to test whether targeted nutritional, prebiotic/probiotic, timing-of-feeding and sleep/circadian interventions during sensitive windows could improve microbial function (SCFAs, bile acids, tryptophan metabolites), sleep architecture, and neurodevelopmental outcomes.

Relevant studies were identified through a PubMed search and selected according to their relevance to the topic for articles published between January 2019 and May 2026, using the following keywords: gut microbiota, gut microbiome, gut flora, gut dysbiosis, sleep, pediatric, child, children, infants, toddlers, adolescents. Article types: Case reports, clinical trials, comparative studies, meta-analysis, observational studies, randomized controlled trials, and reviews.

This review provides an overview of the most recent evidence on the role of the gut microbiota in sleep and sleep disorders in children and summarizes potential microbiota-based therapeutic approaches.

## 2. How Microbiota Can Influence Sleep

The gut microbiota has emerged as a potential regulator of sleep through the microbiota–gut–brain axis, a complex bidirectional network involving neural, endocrine, immune, and metabolic signaling pathways [12,13]. Recent pediatric evidence suggests that gut microbial composition is associated with sleep quality, sleep continuity, and circadian rhythm regulation [14,15].

Higher microbial diversity and enrichment of short-chain fatty acid (SCFA)-producing taxa, particularly members of the Lachnospiraceae family and *Bifidobacterium*, have been associated with more favorable sleep patterns in children, including longer sleep duration and fewer nocturnal awakenings [16]. These associations are biologically plausible, as SCFAs such as butyrate, acetate, and propionate contribute to intestinal barrier integrity, modulation of neuroinflammation, and central nervous system signaling pathways involved in sleep regulation [12]. In contrast, alterations in *Bacteroides* abundance and shifts in the *Firmicutes*/*Bacteroidetes* ratio have been linked to fragmented sleep and circadian disruption [15]. Pediatric studies further suggest that children characterized by “early sleep–early wake” patterns exhibit a more favorable microbial profile, including increased abundance of beneficial taxa such as *Akkermansia* and other SCFA-producing bacteria [15].

Several mechanisms may underlie the relationship between gut microbiota composition and sleep. Gut microbes are involved in tryptophan metabolism and may influence the synthesis of serotonin and melatonin, both of which play essential roles in regulating circadian rhythms and sleep initiation [13]. Moreover, gut dysbiosis may promote systemic inflammation and hypothalamic–pituitary–adrenal axis activation, potentially contributing to sleep disturbances [14].

Although current findings support a link between gut microbiota composition and sleep patterns in pediatric populations, available evidence remains largely observational and heterogeneous. Further longitudinal and mechanistic studies are needed to clarify causality and identify microbial signatures associated with healthy sleep in children.

## 3. Gut–Brain Axis Alterations and Sleep Disorders

An increasing number of studies support a bidirectional relationship between the gut microbiota and sleep disorders in children (Table 1), mediated through the microbiota–gut–brain axis (MGBA) [17]. On one hand, sleep deprivation alters intestinal motility, mucosal integrity, and microbial composition. On the other hand, the microbiota, through neurotransmission, immune–endocrine balance, and inflammatory regulation, can regulate sleep homeostasis and architecture [17,18,19].

Furthermore, recent evidence suggests that modulation of the gut microbiota during early life may reduce the risk and severity of neuropsychiatric disorders, including autism spectrum disorder (ASD), attention-deficit/hyperactivity disorder (ADHD), and anxiety disorders [13].

The gut microbiota is a complex ecosystem composed of trillions of microorganisms (approximately 3.8 × 10^13^) [20] and plays a fundamental role in maintaining physiological homeostasis. Its composition is heavily shaped by several factors acting within the first thousand days of life, including delivery mode, feeding type, use of antibiotics, and environmental factors [13].

Similarly, sleep is also a dynamic biological process, regulated by an integrated network of neural circuits and peripheral signals. The regulation of the sleep–wake cycle involves neurotransmitters produced both centrally and peripherally, as well as endocrine systems such as the hypothalamic–pituitary–adrenal axis [20]. In this context, the gut microbiota can be considered a functional organ capable of producing or modulating neuroactive molecules, including GABA, serotonin, and dopamine, as well as numerous bioactive metabolites [20,21].

In a 2022 study of 162 infants, Schoch et al. suggested a dynamic interaction between the gut microbiota and sleep, mediated by the brain, with potential implications for behavioral and cognitive development during early childhood [18]. Indeed, the gut microbiota can produce or regulate a wide range of metabolites capable of directly or indirectly influencing the nervous system [19].

During childhood, both the gut microbiota and sleep undergo critical stages of maturation [17]. The most significant changes in microbial composition occur in the early years of life, with the gradual establishment of a relatively stable structure that becomes consolidated during puberty [21]. This developmental window represents a period of high plasticity, but also increased vulnerability, making early interventions particularly promising.

### 3.1. Pathophysiological Mechanisms

The gut microbiota communicates with the brain through several pathways, including metabolic [13], neuroendocrine, autonomic, and immunoregulatory mechanisms [21].

#### 3.1.1. Metabolic Pathway

Among the key mediators are short-chain fatty acids (SCFAs), which are produced through the fermentation of dietary fiber by the gut microbiota. Acetate, propionate, and butyrate play a crucial role in maintaining intestinal barrier integrity, promoting mucus production, and modulating serotonin secretion. Some SCFAs have also been detected in the cerebrospinal fluid, suggesting their direct involvement in central nervous system homeostasis and blood–brain barrier integrity [21].

Recent evidence suggests that higher concentrations of propionate are associated with longer sleep duration and fewer night-time awakenings in children [20,22]. Furthermore, SCFAs can influence serotonin production through receptors expressed on enterochromaffin cells and promote GABA synthesis, thereby contributing to the regulation of neuronal excitation and inhibition [21].

In a study of 143 preschool children (mean age, 4.37 ± 0.48 years), Wang et al. identified neuroactive fecal metabolites and immunomodulators associated with improved sleep quality, with propionate being inversely associated with nighttime awakenings [19].

Another key mediator is tryptophan, an essential amino acid metabolized by both the host and the gut microbiota. Its derivatives, including serotonin and melatonin, are essential for regulating the circadian rhythm and sleep quality [19,21].

#### 3.1.2. Neuroendocrine and Autonomic Pathway

The gut microbiota modulates important neuroendocrine systems. GABA, the major inhibitory neurotransmitter, promotes sleep and regulates the stress response, and certain bacterial strains are capable of producing it directly. At the same time, serotonin, produced largely in the gut, is the precursor of melatonin, a key hormone in the regulation of the sleep–wake cycle [19,20,23].

Melatonin, secreted not only by the pineal gland but also by the gut, exerts systemic and local effects, contributing to the regulation of circadian rhythms and antioxidant protection. Alterations in the microbiota and sleep deprivation can reduce its levels, further compromising sleep architecture [20].

The hypothalamic–pituitary–adrenal (HPA) axis also plays a central role [20]. Its hyperactivation, with increased cortisol levels, is associated with sleep fragmentation, a reduction in deep sleep stages, and an increase in awakenings [24]. Sleep deprivation can, in turn, further amplify this response, creating a vicious cycle.

Finally, the vagus nerve acts as a direct communication pathway between the gut and the brain, transmitting neurochemical and inflammatory signals capable of influencing sleep regulation [20].

#### 3.1.3. Immunological Pathway

The gastrointestinal tract is the body’s primary immune organ [24]. The intestinal barrier plays a crucial role in preventing potentially harmful substances from entering the systemic circulation [20]. However, in the presence of dysbiosis, intestinal permeability may increase, leading to bacterial translocation and activation of a systemic inflammatory response [25].

Pro-inflammatory cytokines such as IL-1β, IL-6, and TNF-α can cross the blood–brain barrier and alter neuronal excitability, negatively affecting sleep. SCFAs and tryptophan metabolites, by contrast, exert anti-inflammatory and neuroprotective effects by modulating microglial activation [17].

Emerging evidence indicates that the gut microbiota also exhibits circadian rhythmicity, which develops progressively during childhood. The synchronisation between microbial rhythms and the sleep–wake cycle contributes to the maintenance of metabolic homeostasis.

Recent studies have shown that different sleep patterns (early vs. late chronotypes) are associated with specific microbial and metabolic profiles. In particular, going to bed late appears to be linked to a reduction in microbiota diversity and an increase in potentially harmful metabolites [15,26].

In the study by Mao et al. [15] conducted on 88 children between 2 and 14 years of age, the metabolic pathway of those who go to bed early and get up early and those who go to bed late and get up late was analyzed, and it was shown that falling asleep time is associated with the microbiota. Sleep timing is associated with distinct microbial profiles, suggesting an interaction between the sleep–wake cycle, the gut microbiota, and metabolic homeostasis. Another study on the same sample written by Xiang et al. [26] revealed significant differences between plasma and fecal metabolites in children with early or late sleep schedules, suggesting that going to bed late may decrease the diversity, richness, and function of the gut microbiota and increase unwanted microbial metabolites.

### 3.2. Neurodevelopmental Disorders, Gut Microbiota, and Sleep

Neurodevelopmental disorders represent an important clinical setting in which alterations of the microbiota–gut–brain axis and sleep disturbances frequently coexist. Emerging evidence suggests that gut microbial dysbiosis may contribute to neurodevelopmental and behavioral abnormalities through immune, metabolic, endocrine, and neural pathways, potentially affecting sleep regulation as well [13].

Autism spectrum disorder (ASD) has been consistently associated with gastrointestinal dysfunction and alterations in gut microbial composition [27]. Dysbiosis may influence the production of neuroactive metabolites, including short-chain fatty acids and tryptophan-derived compounds involved in serotonin and melatonin metabolism, both of which play key roles in sleep regulation and circadian rhythm maintenance. Sleep disturbances are among the most common comorbidities in children with ASD and may further exacerbate behavioral and cognitive difficulties [13]. Similarly, children with attention-deficit/hyperactivity disorder (ADHD) frequently experience sleep disturbances, including difficulties initiating and maintaining sleep, reduced sleep duration, and alterations in circadian rhythms. Recent studies suggest that microbiota-related mechanisms may influence neurotransmitter systems involved in both attention regulation and sleep–wake regulation, supporting a potential role of the gut microbiota in the pathophysiology of ADHD [13].

Anxiety symptoms and emotional dysregulation have also been associated with alterations in the gut microbiota through mechanisms involving immune activation, hypothalamic–pituitary–adrenal axis dysregulation, and altered microbial metabolite production. Since anxiety disorders are frequently associated with poor sleep quality, insomnia, and fragmented sleep, microbiota-mediated pathways may represent a common biological substrate connecting emotional and sleep disturbances [13].

Overall, neurodevelopmental disorders provide a relevant model for understanding the complex interactions among gut microbiota, sleep, and brain function during childhood. Further longitudinal studies are needed to clarify causal relationships and evaluate whether microbiota-targeted interventions may improve both sleep and neurobehavioral outcomes in pediatric populations.

A particularly relevant aspect of pediatric populations is that sleep architecture, circadian regulation, gut microbiota composition, and neurodevelopment undergo simultaneous maturation during infancy and childhood [4,18]. Therefore, alterations of the microbiota–gut–brain axis during these critical developmental windows may have broader consequences than those observed in adults, potentially affecting not only sleep regulation but also cognitive, behavioral, and emotional development [4,13]. Across pediatric studies, some findings appear relatively consistent, including the association between favorable sleep patterns, greater microbial diversity, and increased abundance of SCFA-producing bacteria and metabolites [15,19,22]. However, several questions remain unresolved. Compared with adults, pediatric data on specific sleep disorders, including insomnia, obstructive sleep apnea, circadian rhythm disorders, and sleep disturbances associated with neurodevelopmental conditions, remain limited [2,13]. Moreover, it is still unclear whether microbiota alterations represent a cause or a consequence of sleep disturbances and whether the mechanisms identified in adults can be directly extrapolated to pediatric populations [2,17].

**Table 1:** Pediatric studies investigating the association between gut microbiota and sleep.

Study	Population	Study Design	Sleep Outcome Assessed	Main Microbiota Findings	Key Findings
Schoch et al., 2022 [18]	162 infants during the first year of life	Longitudinal cohort study	Sleep development, sleep–wake maturation	Dynamic interaction between microbial maturation and brain development	Demonstrated a bidirectional relationship among gut microbiota, sleep, and neurodevelopment during infancy.
Heath et al., 2020 [22]	Infants	Observational study	Sleep duration and night awakenings	Higher fecal propionate concentrations associated with longer sleep duration and fewer awakenings	SCFAs may contribute to healthy sleep regulation in early life.
Wang et al., 2022 [19]	143 preschool children (mean age 4.37 ± 0.48 years)	Cross-sectional study	Sleep quality and nighttime awakenings	Neuroactive metabolites and SCFAs correlated with sleep quality; propionate inversely associated with nocturnal awakenings	Gut microbial metabolites may influence sleep quality through microbiota–gut–brain signaling.
Mao et al., 2024 [15]	88 children aged 2–14 years	Cross-sectional study	Sleep timing and chronotype	Distinct microbial communities according to sleep timing	Sleep timing is significantly associated with gut microbial composition and metabolic pathways.
Xiang et al., 2023 [26]	Children from Northwest China	Multi-omics observational study	Early versus late sleep schedules	Reduced microbial diversity and altered metabolomic profiles in late sleepers	Late sleep behavior may negatively affect microbiota richness and metabolic homeostasis.

## 4. Modulating Dysbiosis: Interventional Studies

Based on the available evidence, promoting the development of a healthy gut microbiota during the first year of life may have long-term beneficial effects on children’s health, whereas dysbiosis may adversely affect sleep regulation, brain connectivity, and neurobehavioral development. Accordingly, the administration of prebiotics and probiotics may support gut microbiota maturation and improve sleep and neurocognitive outcomes [28].

A fiber-rich diet is fermented by the gut microbiota in the colon, leading to the production of SCFAs such as acetate, propionate, and butyrate, which play key roles in immune regulation, modulation of inflammation, and maintenance of intestinal barrier integrity [24]. These metabolites may improve sleep quality by modulating the synthesis of serotonin, GABA, and, consequently, melatonin, thereby contributing to sleep regulation and circadian function [21].

Therefore, increased dietary fiber intake may influence sleep through several mechanisms, including enhancing microbial diversity, reducing inflammation and intestinal permeability, and promoting serotonin production [24].

Nutrition, including dietary patterns, meal timing, and meal regularity, has a significant impact on gut microbiota composition. Therefore, a balanced diet rich in healthy fats and dietary fiber is essential for maintaining a healthy gut microbiota and, consequently, promoting good sleep quality [21].

Several experimental studies investigating the effects of diet, prebiotics, and probiotics on sleep have been conducted in animal models (Table 2). Thompson et al. demonstrated in young rats that a diet rich in prebiotics and bioactive milk components improved NREM sleep quality, enhanced REM sleep recovery after stress, and preserved gut microbiota composition, suggesting that nutrition may improve sleep and stress resilience through modulation of the gut microbiota [29]. This is confirmed by a further study by Bowers et al., also on young rats administered a diet containing prebiotics with a sleep interruption protocol subsequently applied. The results showed that the prebiotic diet significantly changed the composition of the microbiota and that during the sleep disruption protocol, rats treated with the prebiotic diet showed greater resistance to the effects of sleep deprivation and after the deprivation phase showed longer total sleep duration and longer and more stable NREM sleep episodes [30].

Only a limited number of studies have investigated the effects of prebiotics on behavior and sleep in children. Colombo et al. published a randomized, double-blind study conducted on 161 infants; some received formula milk, others formula milk with the addition of a mix of prebiotics (polydextrose and galactooligosaccharides). Children receiving the prebiotic blend exhibited greater beta diversity and a significantly higher abundance of beneficial bacterial taxa than those in the control group. Prebiotic supplementation was associated with earlier maturation of physiological sleep–wake patterns [31].

Zimmermann et al. proposed a randomized, double-blind, placebo-controlled study of 120 preterm and 260 full-term children who were administered a placebo or synbiotics daily for 3 months to assess the outcome in terms of neurodevelopment and sleep patterns at 2 years of life [28]. Data collection involves studying the composition of the intestinal microbiota through metagenomic analysis, assessing sleep patterns through questionnaires, actigraphy, and high-density EEG, analyzing intestinal and breath metabolites, and assessing the outcome related to neurobehavioral development using standardized tools. The findings could lead to new early intervention strategies for newborns, especially those born prematurely or at risk for neurobehavioral problems. The study is ongoing (enlistment plan 2025–2029). Despite not publishing the full results, the protocol has been ethically approved and registered as an international clinical trial [28].

These findings suggest that identifying effective interventions targeting the gut–brain axis during early childhood may have long-term positive implications for children’s health and development [28].

The main microbiota-based therapeutic strategies include probiotics, such as *Bifidobacterium* and *Lactobacillus* species, which may reduce hypothalamic–pituitary–adrenal axis activation and inflammation; prebiotics, which promote the growth of beneficial bacteria, improve metabolite production, support microbiota balance, and may enhance sleep quality; and fecal microbiota transplantation, which has been associated with reduced sleep latency, increased sleep duration, and improved quality of life in patients with sleep disorders. Emerging approaches also include modulation of microbial metabolites, anti-inflammatory therapies, nutritional interventions, and circadian rhythm-based strategies [17].

The limitations of current evidence are due to the paucity of studies specific to pediatric age, methodological heterogeneity, sometimes the subjectivity of sleep measures, the current lack of standardization of study protocols, and, consequently, a reduced ability to establish causal relationships.

Future research should focus on identifying specific probiotic strains, defining optimal dosages, and developing personalized microbiota-based interventions through well-designed longitudinal studies evaluating long-term outcomes.

**Table 2:** Microbiota-based interventions targeting sleep and neurodevelopment.

Study (Ref.)	Population	Intervention	Study Design	Main Microbiota Effects	Main Sleep-Related Outcomes
Thompson et al. [29]	Young rats	Prebiotic-rich diet containing bioactive milk fractions	Experimental study	Preservation of microbial alpha diversity and protection against stress-induced microbial alterations	Improved NREM sleep quality, enhanced REM sleep rebound after stress exposure
Bowers et al. [30]	Young rats subjected to sleep disruption	Prebiotic diet	Experimental study	Significant remodeling of gut microbiota composition and increased resilience to dysbiosis	Greater resistance to sleep disruption, longer total sleep duration, and more stable NREM sleep episodes
Colombo et al. [31]	161 healthy infants	Infant formula supplemented with polydextrose and galactooligosaccharides (prebiotic blend)	Randomized double-blind controlled trial	Increased beta diversity and higher abundance of beneficial bacterial taxa compared with controls	Faster maturation of physiological sleep–wake patterns

## 5. Conclusions and Future Perspectives

Overall, microbiota-based therapeutic strategies may represent safe and potentially effective adjunctive therapies for several neurological disorders. In particular, evidence from human studies links sleep disturbances and insomnia to gut microbial dysbiosis, characterized by reduced microbial richness, depletion of butyrate-producing genera (*Faecalibacterium* and *Lachnospira*), and enrichment of *Blautia* and *Eubacterium hallii*. These alterations have been associated with insomnia severity, pro-inflammatory markers, and metabolic changes [2,4,11]. Evidence indicates that, during early life, feeding practices, maternal nutrition, and perinatal exposures shape microbial maturation and are associated with nap patterns, sleep consolidation, and early cognitive outcomes. Nutritional interventions, including DHA, choline, iron, iodine, vitamin B12, vitamin D, HMOs, and probiotics, have shown promising preliminary effects on both microbial composition and sleep-related outcomes [2,4].

Despite the promising mechanistic evidence, including reduced SCFA production, altered tryptophan–melatonin metabolism, immune activation, and HPA axis/circadian disruption, pediatric-specific causal evidence remains limited, and more robust clinical studies are needed before definitive conclusions can be drawn.

In the coming years, well-designed clinical studies, including pediatric longitudinal multi-omics cohorts and randomized controlled trials evaluating prebiotic and probiotic interventions during critical developmental windows, will be essential to clarify the therapeutic potential of microbiota-based interventions for pediatric sleep disorders.

## Data Availability

No new data were created in this study.
